# Fucoidan from *Fucus vesiculosus* Inhibits Inflammatory Response, Both In Vitro and In Vivo

**DOI:** 10.3390/md21050302

**Published:** 2023-05-17

**Authors:** Lingzhi Wang, Catarina Oliveira, Qiu Li, Andreia S. Ferreira, Cláudia Nunes, Manuel A. Coimbra, Rui L. Reis, Albino Martins, Chunming Wang, Tiago H. Silva, Yanxian Feng

**Affiliations:** 1 School of Biotechnology and Health Sciences, Wuyi University, Jiangmen 529000, China; 2 3B’s Research Group, I3Bs—Research Institute on Biomaterials, Biodegradables and Biomimetics, University of Minho, Headquarters of the European Institute of Excellence on Tissue Engineering and Regenerative Medicine, AvePark, Parque de Ciência e Tecnologia, Zona Industrial da Gandra, Barco, 4805-017 Guimarães, Portugal; 3 ICVS/3B’s–PT Government Associate Laboratory, Braga, 4710-057 Guimarães, Portugal; 4 State Key Laboratory of Quality Research in Chinese Medicine, Institute of Chinese Medical Sciences, University of Macau, Macau, China; 5 LAQV-REQUIMTE, Department of Chemistry, University of Aveiro, Campus de Santiago, 3810-193 Aveiro, Portugal; 6 CICECO, Department of Chemistry, University of Aveiro, Campus de Santiago, 3810-193 Aveiro, Portugal

**Keywords:** fucoidan, *Fucus vesiculosus*, anti-inflammatory, macrophage

## Abstract

Fucoidan has been reported to present diverse bioactivities, but each extract has specific features from which a particular biological activity, such as immunomodulation, must be confirmed. In this study a commercially available pharmaceutical-grade fucoidan extracted from *Fucus vesiculosus*, FE, was characterized and its anti-inflammatory potential was investigated. Fucose was the main monosaccharide (90 mol%) present in the studied FE, followed by uronic acids, galactose, and xylose that were present at similar values (3.8–2.4 mol%). FE showed a molecular weight of 70 kDa and a sulfate content of around 10%. The expression of cytokines by mouse bone-marrow-derived macrophages (BMDMs) revealed that the addition of FE upregulated the expression of CD206 and IL-10 by about 28 and 22 fold, respectively, in respect to control. This was corroborated in a stimulated pro-inflammatory situation, with the higher expression (60 fold) of iNOS being almost completely reversed by the addition of FE. FE was also capable of reverse LPS-caused inflammation in an in vivo mouse model, including by reducing macrophage activation by LPS from 41% of positive CD11C to 9% upon fucoidan injection. Taken together, the potential of FE as an anti-inflammatory agent was validated, both in vitro and in vivo.

## 1. Introduction

Marine-origin byproducts may present an interesting source of unexplored anti-inflammatory compounds such as polyphenols, proteins, and sulfated polysaccharides (e.g., fucoidan). These marine-derived bioactive compounds may constitute interesting alternatives to currently available synthetic drugs, having potential protective effects over the pathogenesis of inflammatory diseases and lower side effects [1]. Fucoidan is a sulfated polysaccharide mainly composed of fucose units and other carbohydrate monomers as minor components [2]. It can be extracted from different species of brown algae, *Fucus vesiculosus* being the most reported and well-studied [3]. Fucoidan may present different biological activities such as antitumor, antiviral, anti-angiogenic, and anti-inflammatory [4,5,6,7]. Different structural characteristics have been associated with the biological activity of fucoidans, molecular weight and sulfation degree being the most described ones [8]. More recent studies have also related the bioactivities of fucoidan to sulfates position and branching degree [9,10].

Fucoidan is being investigated for short- and long-term inflammation treatment [11,12]. The anti-inflammatory mechanisms of action of fucoidans comprise antioxidant, transcription factors, adhesion molecules, matrix metalloproteinases, complement cascade properties, and it can also regulate pro-inflammatory enzymes and the expression of related genes [7]. The most discussed possible mechanism of action is the downregulation of MAPK and NF-κB signaling pathways, followed by a decrease in pro-inflammatory cytokines [12]. Different in vitro studies showed promising anti-inflammatory results for fucoidan from *Fucus vesiculosus.* For example, fucoidan decrease TNF-α and IL-1β levels in mouse macrophages (RAW 264.7 cell line) stimulated by lipopolysaccharide (LPS) [13]. The immunomodulatory properties of photopolymerizable fucoidan were also evaluated in human monocytes (THP-1 cell line) differentiated into macrophages, presenting similar activity as IL-10, by decreasing LPS- and IFN-γ-induced CD86 expression [14]. In LPS-stimulated BV2 microglia cells, fucoidan inhibited the production of nitric oxide (NO) and prostaglandin E2 (PGE2), and reduced the expression of inducible nitric oxide synthase (iNOS), IL-1β, and TNF-α, for example [15]. These anti-inflammatory properties of fucoidan were achieved by the suppression of NF-κB, MAPK, and AKT molecular pathways. A decreased production of pro-inflammatory cytokines (e.g., TNF-α, IL1β, IL-6) was also observed when human keratinocytes and Caco-2 cells were treated with fucoidan [16,17]. Likewise, the inhibition of COX enzymes and hyaluronidase by fucoidan have been observed in a concentration-dependent manner, similarly to the expression of MAPK p38 by LPS-stimulated human mononuclear U937 cells [18]. Recently, it has been proposed by Obluchinskaya et al. that fucoidan inhibited the protein denaturation observed in inflammation, with the effect depending on fucoidan concentration and being mainly associated with fucose contents, although the sulfate content may also play a role [19]. Fucoidan from *Fucus vesiculosus* has decreased neutrophil infiltration, as well as systemic inflammation, presenting lower levels of TNF-α and IL1β in mice [20]. Similarly, a fucoidan extract from the same species inhibited the recruitment of leucocytes in an inflammation rat model, apparently in a way not dependent on fucoidan’s chemical structure, but without association to P-selectin, in opposition to fucoidans from other brown algae species [21]. Fucoidan is also capable of reducing the inflammation (decreased levels of IL-1α, IL-1β, and IL-10 of bowel disease when orally administered [22]. Moreover, a fucoidan-based cream was capable of inhibiting carrageenan-induced edema in rats upon topical application in a dose-dependent manner, being comparable to a diclofenac gel [23].

All these studies were carried out using purified fucoidans, allowing for the acquisition of structure–function relationships. However, the term “fucoidan” encloses a group of polysaccharides composed of a fucose backbone chain, often sulfated, with a range of chemical characteristics (molecular weight, sugar profile, sulfation degree and pattern, among others), depending on the specific seaweed sample and extraction methodology [24]. Thus, the production of large-scale and commercially available fucoidan extracts, even at a pharmaceutical grade, hardly allows obtaining a unique bioactive structure, and different fucoidan extracts can correspond to different (groups of) chemical entities, which could prevent its anti-inflammatory properties. In this study, we selected a specific commercially available fucoidan extract from *Fucus vesiculosus*, herein represented as FE, hypothesizing that it contained the active structures able to influence in vitro and in vivo inflammation. Therefore, FE was chemically characterized, and its potential anti-inflammatory activity was assessed both in vitro using the RAW 264.7 cell line and bone-marrow-derived macrophages, and in vivo upon intraperitoneal injection in mice.

## 2. Results and Discussion

### 2.1. Structural Characterization of Fucoidan Extract (FE)

Molecular weight and sulfation degree are the most described physicochemical factors influencing fucoidan bioactivity [8]. Moreover, monosaccharide composition and sulfate position may also influence the final biological activity, recently being taken into consideration [9]. The brown seaweed species and specific reproductive phase of the used sample, as well as the extraction method, may be the possible triggers to influence these intrinsic properties [3,25,26]. Given this variability, with the structure–activity relationship not yet fully established, the biological activity of each new extract being produced should not be assumed, but confirmed.

Taking this into consideration, the fucoidan extract (FE), herein studied, was extensively characterized. The total carbohydrate content of FE was 66% (*w/w*) and had a sulfation degree of around 10% (Table 1). The monosaccharide composition was assessed, confirming that fucose was the main sugar (around 90% mol) (Table 1), with uronic acids (3.8 mol%), galactose (3.3 mol%), and xylose (2.4 mol%) present in smaller amounts. FE had a molecular weight of 70 kDa and a polydispersity (Mw/Mn) of 1.5, confirmed by the gel permeation chromatography (GPC) analysis (Table 1).

Due to the different fucoidan species and extraction methods, it is difficult to define specific and strict ranges for the parameters described above to meet the biological responses. The molecular weight of FE herein studied was within the range that others reported in the literature, from few kDa to over 100 kDa [3,10]. The total of carbohydrate content was slightly increased in this extract when compared to other extracts that have been previously characterized by our group (50–52.5% *w/w*) and the sulfation degree was lower (around 30% in the other extracts) [9]. Furthermore, this FE presented a higher proportion of Fuc and a lower proportion of other monosaccharides when compared to those same extracts (fucose 71.2–79.1% mol, uronic acids 9.8–15.3% mol, xylose 3.9–8.0% mol, and galactose 3.5–5.5% mol). These differences may be attributed to the purity of this FE, since the polysaccharide extract herein used was a pharmaceutical grade, with a purity of 98%.

To determine the type of glycosidic linkages and the sulfate group position along the polymer chain of FE, a methylation analysis was performed before and after desulfation. If a position is acetylated in the native polysaccharide and becomes methylated after desulfation, it is an indication of a sulfate residue at that position. Thus, FE was mainly sulfated in C-2 and C-4, since the glycosidic linkages 2,3,4-Fuc and 2,4-Fuc decreased after desulfation, and 3,4-Fuc, 2-Fuc, 3-Fuc, and 4-Fuc increased (Table 2). The residue of 2,3-Fuc was not disadvantaged by significant changes and, consequently, FE could be branched at C-2, with the linkage C-3 belonging to the main chain. The residues of the other sugars (t-Xyl, 2-Xyl, and t-Gal) did not show relevant modification in the residue content for native and desulfated FE. This procedure was performed to better understand the level of polymer branching and position of sulfate groups in fucose. The fine characterization of the chemical structure might require the chromatographic purification of FE to obtain fractions composed by (ideally) single chemical components, which is beyond the goal of the present study.

### 2.2. Toxicity of the Fucoidan Extract (FE) over RAW 264.7 Macrophages

**Figure 1:**
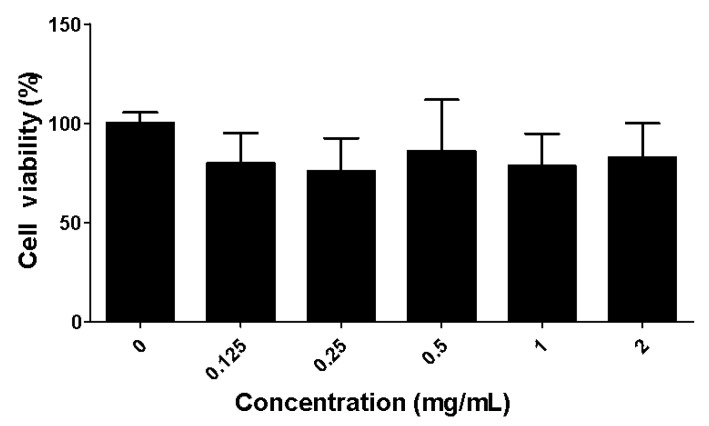
Assessment of polysaccharide cytotoxicity in RAW 264.7 murine macrophages. Cell viability was quantified with CCK-8 assay after incubation with FE for 48 h.

RAW 264.7 murine macrophage cell line is often used as a first screening model of natural products’ bioactivity and to predict their potential effect [22]. The effects of FE on the viability of RAW 264.7 macrophages were assessed for different FE concentrations after 48 h of incubation (Figure 1). There is no positive correlation between cell viability and FE concentrations, and no significant differences were observed for all tested conditions. All further experiments were conducted using 0.1 mg/mL, where negligible cytotoxic effects were observed, since macrophage viability was above 70% (in accordance with ISO 10993-5). This same range of fucoidan concentrations (0.1 mg/mL) did not impair macrophage’ growth, as previously reported by others [13,23].

### 2.3. Expression of Pro- and Anti-Inflammatory Cytokines

Mouse bone-marrow-derived macrophages (BMDMs) are a kind of primary cells, extracted directly from the alive animal. Although RAW 264.7 cells are frequently used as models to assess (anti-)inflammatory response because they are widely available and easy to culture, evidence has revealed that the main biological process involving the cell cycle control, cytoskeleton reorganization, and apoptosis was significantly dissimilar from RAW 264.7 cells to BMDM [27]. Alongside that, BMDMs are proven to be more sensitive and a more realistic response to an inflammatory stimulus [28], thus being selected as the in vitro model in this study. After 48 h incubation with FE, different cytokines (TNF-α and IL-12 as pro-inflammatory, and CD206 and IL-10 as anti-inflammatory) were quantified [29]. TNF-α activates different cell signaling pathways and mediates the production of many other inflammatory mediators, participating in the initiation and progression of the inflammatory response [30]. IL-12 is produced early during infections, and comprises a heavy chain (p. 40, [31]) and a light chain (p. 35, [31]). This cytokine is associated with innate and adaptative immunity through the induction of IFN-γ. IL-10 is produced via a wide variety of activated immune cells, and its main actions are anti-inflammatory, inhibitory, or self-regulating [32]. CD206, a mannose receptor, is a membrane-bound protein, predominantly expressed by macrophages and dendritic cells, acts as a pattern recognition receptor that plays a role in innate and adaptive immunity [33,34].

**Figure 2:**
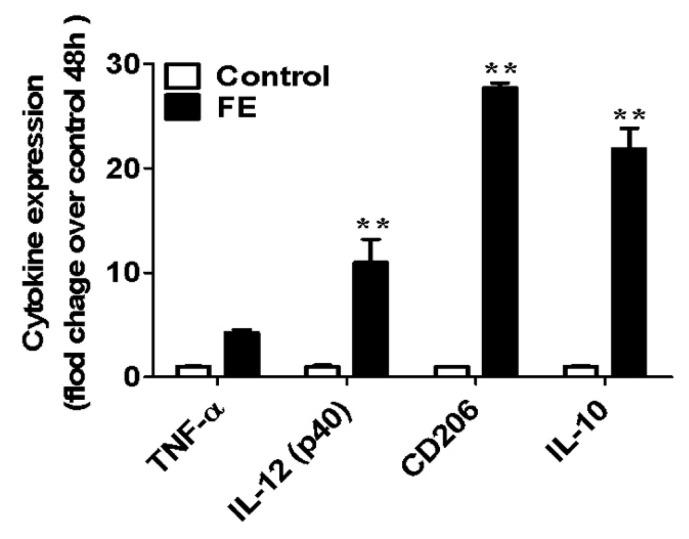
Gene expression of pro- (TNF-α and IL-12) and anti-inflammatory (CD-206 and IL-10) cytokines. Significance was set to a *p*-value < 0.05. Results are presented as the mean ± standard deviation; ** *p* < 0.01.

The effect of FE on the expression of the above-mentioned cytokines by BMDM was studied (Figure 2). The presence of 100 µg/mL FE induced a slight increase in TNF-α expression with no statistically significant differences compared to the control condition. However, the expression of IL-12, CD206 and IL-10 was significantly higher than the control condition. Indeed, the expression of CD206 and IL-10 was much higher than the other two quantified cytokines, demonstrating the anti-inflammatory potential of FE.

### 2.4. Reversed Inflammation in M1 Phenotype

**Figure 3:**
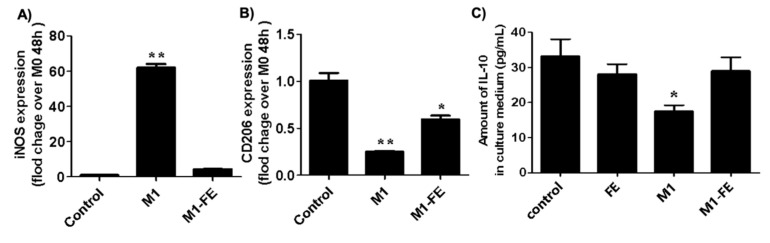
Expression of iNOS (**A**) and CD206 (**B**) determined via RT-qPCR, and the amount of IL-10 quantified by ELISA (**C**); all inflammatory molecules synthesized by BMDM, stimulated or not stimulated by LPS and IFN-γ for 12 h, and after FE incubation for 48 h. Significance was set to a *p*-value < 0.05. Results are presented as the mean ± standard deviation; * *p* < 0.05, ** *p* < 0.01.

Inflammatory stimuli such as LPS (lipopolysaccharides) induces macrophage activation and, consequently, the expression of cytokines that mediates different stages of inflammation [35,36]. Indeed, the inhibition or induction of cytokine production plays a crucial role in the control of inflammation. To assess the effects of FE over the expression of anti- and pro-inflammatory molecules such as inducible nitric oxide synthase (iNOs), CD206, and IL-10 in a simulated inflammatory situation, BMDMs were subject to LPS+IFN-γ stimulation for 12 h. iNOS is one of the direct consequences of an inflammatory process and a major mediator of inflammation in various cell types [37]. As observed in Figure 3A, the expression of iNOS was significantly increased after LPS+IFN-γ stimulation, as expected. This situation was reversed through the addition of FE, with expression levels similar to the basal condition. The addition of FE increased CD206 expression in response to LPS+IFN-γ stimulation. A similar behavior was observed for IL-10 protein, another anti-inflammatory cytokine. Previous studies reported the effect of fucoidan from *Fucus vesiculosus* at similar concentrations (i.e., 50–100 µg/mL) on different cell types, specifically RAW 264.7, Caco-2, and BV2 microglial cells [13,15,17]. These studies showed that fucoidan decreased the expression of pro-inflammatory cytokines after LPS-stimulation. Fucoidan from species other than *Fucus vesiculosus* also presented some promising results. Fucoidan extracts from *Laminaria japonica* reduced the expression of iNOS, TNF-α, and IL-6 [38,39]. In a particular study, Caco-2 cells were used, and both pro- and anti-inflammatory cytokine expressions were assessed [40]. A decreased expression of TNF-α and IL-1β, along with increased levels of IL-10 and IFN-γ, was observed for fucoidan from *Sargassum hemiphyllum*.

Herein, a decreased expression of pro-inflammatory and an increase in anti-inflammatory mediators validate the anti-inflammatory potential of FE in vitro, after settling a simulated inflammatory situation. These observations state the reversed inflammation capacity of this fucoidan extract (FE).

### 2.5. FE Anti-Inflammatory Response in a Mouse Model

**Figure 4:**
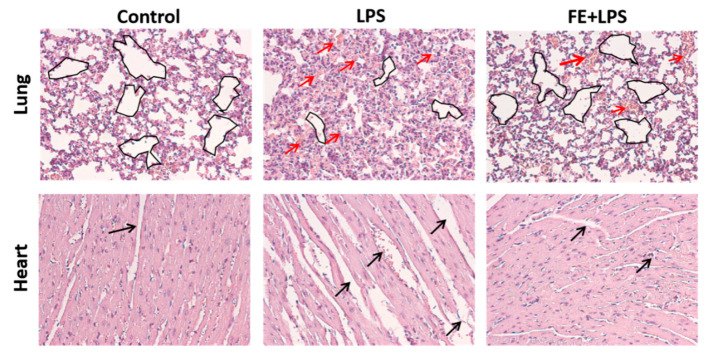
Morphology of inner organ (lung and heart) stained by H&E. Red (lung) and black (heart) arrows highlight morphological changes in the tissues, more evident in the group treated with LPS but mostly reversed when FE was also injected.

The anti-inflammatory potential of FE was also assessed in vivo through the intraperitoneal injection of LPS in a mouse model. Histologically, the typical features of normal cardiac and lung tissues, or mild morphological changes, were observed in mice injected with FE after LPS stimulation (Figure 4).

**Figure 5:**
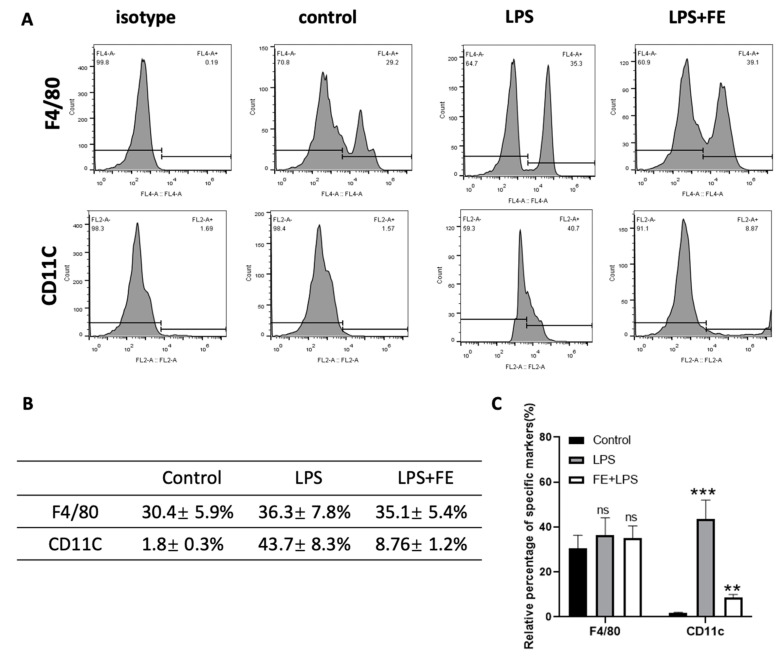
Flow cytometry analysis of F4/80 and CD11C expressed by peritoneal macrophages. (**A**) Flow cytometry histograms. (**B**) Relative percentage of F4/80 and CD11C markers. (**C**) Statistic analysis of specifically marked cells; ns—difference not statistically significant, ** *p* < 0.01, *** *p* < 0.001.

These observations are in agreement with the ones of another study, whereby fucoidan administration attenuated myocardial damage [28]. To ascertain the influence of FE over tissue-specific macrophages, intraperitoneal macrophages were marked for the surface markers F4/80 and CD11C (Figure 5). Flow cytometry analysis showed that the F4/80 marker is highly expressed upon FE administration. Indeed, tissue-specific macrophages aid in maintaining homeostasis and triggering the immune system in response to a stimulus [41,42]. Oppositely, the integrin CD11C that is highly expressed in monocytes and macrophages, was downregulated when FE was injected, presenting values similar to the control condition (healthy mice). Therefore, FE did not evoke a CD11C-mediated response, which is an indicator of macrophage activation [43]. These observations indicate that the administration of FE induced a decrease in the inflammatory response upon LPS stimulation.

## 3. Materials and Methods

### 3.1. Materials

A pharmaceutical-grade fucoidan extract from Marinova (batch number—DPFVF2015505), herein referred to as FE, with a purity of 98%, was purchased and used as received. Lipopolysaccharides (LPS from *E. coli* 055:B5) were purchased from InvivoGen Co., Ltd. (San Diego, CA, USA). Enzyme-linked immunosorbent assay (EISA) kits for murine IL-10 were purchased from NeoBioscience Co., Ltd. (Shenzhen, China). FITC anti-mouse F4/80 antibody and APC anti-mouse CD11C antibody for flow cytometric analysis were purchased from Biolegend (San Diego, CA, USA). Recombinant murine IFN-γ was purchased from Peprotech (Cranbury, NJ, USA). The mouse macrophage cell line Raw 264.7 was purchased from American-Type Culture Collection (ATCC, Manassas, VA, USA), and maintained according to the culture guidelines. All reagents used for the cell culture were purchased from Gibco Life Technology (Carlsbad, CA, USA).

### 3.2. FE Characterization

#### 3.2.1. Molecular Weight

FE molecular weight was determined by GPC using a methodology previously described [9]. Briefly, a Malvern Viscotek TDA 305 (Malvern, UK) system composed of a set of four columns: pre-column Suprema 5 µm 8 × 50 S/N 3111265, Suprema 30 Å 5 µm 8 × 300 S/N 3112751, Suprema 1000 Å 5 µm 8 × 300 S/N 3,112,851 PL, and Aquagel-OH MIXED 8 µm 7.5 × 300 S/N 8M-AOHMIX-46-51, followed by a refractometer (RI-Detector 8110, Bischoff), right-angle light scattering, and viscometer detectors. For the eluent, a solution of 0.1 M NaN_3_ and 0.01 M NaH_2_PO_4_ with a pH = 6.6 was used at a flow rate of 1 mL min^−1^, and the system kept at 30 °C. To calibrate elution times, a commercial set (Varian ^®^, Palo Alto, CA, USA) of pullulans with narrow polydispersity and Mp (molecular mass at the maximum chromatographic peak) varying from 0.18 to 708 kDa was used.

#### 3.2.2. Sulfate Content

The sulfate ester content present in FE was determined via elemental analysis using a Truspec 630-200-200 with a TCD detector, with 2 mg of each sample in duplicate. The temperatures were set at 1075 °C (combustion furnace) and 850 °C (after burner temperature). The sulfur value obtained was converted to the sulfate ester content of the FE, using a calculation methodology adapted from [44,45].

#### 3.2.3. Monosaccharide Composition

Neutral monosaccharides were determined as alditol acetates via gas chromatography with a flame ionization detector (GC-FID), using 2-deoxyglucose as the internal standard, as described elsewhere [46]. Briefly, pre-hydrolysis with 72% sulfuric acid was performed on the FE for 3 h at room temperature (RT), followed by a 2.5 h hydrolysis at 100 °C with 1 M sulfuric acid. The obtained monosaccharides were reduced with sodium borohydride and acetylated with acetic anhydride using methylimidazole as the catalyst.

A modification of the colorimetric method 3-phenylphenol was used to quantify the uronic acids [46]. Samples were hydrolyzed with 1 M sulfuric acid at 100 °C for 1 h. Galacturonic acid was used to make an external calibration curve. Hydrolysis was performed in duplicate for all samples. A third analysis was performed concerning the samples with higher variability.

The total content of carbohydrates was determined through the sum of the monosaccharide contents.

#### 3.2.4. Glycosidic Linkage and Substitution Analysis

Methylation analysis was used to determine the glycosidic linkages and the position of sulfate groups [46].

For desulfation, dimethyl sulfoxide (1.8 mL) was used to dissolve the samples (10 mg), followed by the addition of pyridine (0.1 mL), pyromellitic acid (13 mg), NaF (12 mg), and pyridine (0.2 mL), in a sequential order. This solution was stirred at 120 °C for 3 h, cooled, and poured into a NaHCO_3_ solution (1 mL). The desulfated polysaccharide solution was dialyzed and freeze-dried. Methylation analysis was performed on the desulfated polysaccharides.

Anhydrous dimethylsulfoxide (1 mL) was used to dissolve native and desulfated samples (1–2 mg), and 40 mg NaOH was added in an argon atmosphere. The methylation was performed by stirring the samples for 20 min in 80 μL CH_3_I, which was added two more times. This was followed by the addition of a mixture of CHCl_3_/MeOH (1:1, *v/v*, 3 mL), and dialyzed (12–14 kDa) against 50% EtOH. A 2 M TFA solution at 120 °C for 1 h was used to hydrolyze the methylated sample and, after that, the sample was reduced and acetylated, as previously described for neutral sugar analysis (using NaBD_4_ instead of NaBH_4_). Gas chromatography coupled with mass spectrometry (GC-qMS) was used to separate and analyze the partially methylated alditol acetates.

### 3.3. Biological Assays

#### 3.3.1. Assessment of Cytotoxic Effects of FE on the RAW264.7 Cell Line

RAW 264.7 cells were seeded onto 96-well plates (5000 cells per well). After 12 h, the cells were co-incubated with various concentrations (0.125, 0.25, 0.5, 1, 2 mg/mL) of FE. After another 48 h, the cell viability was tested according to Cell Counting Kit-8 (CCK-8), whereby the reaction reagent, WST-8 (10 μL), was added into each well and incubated for 2 h. The absorbance was measured at 450 nm.

#### 3.3.2. Assessment of the Pro-/Anti-Inflammation Effects of FE on Mouse Bone-Marrow-Derived Macrophages

Mouse bone-marrow-derived macrophages (BMDMs) were extracted from the back limbs of mice and cultured according to previous studies. The primary BMDMs were seeded on a six-well culture plate (2 × 10^6^ cells per well) for direct use, or pre-induced into a M1 phenotype and treated with LPS (100 ng/mL) and IFN-γ (40 ng/mL) for 12 h. After co-incubated with FEs (100 μg/mL) for 24 h, the cells were gently washed with PBS, and their RNA was collected with TRIzol for subsequent RT-qRCR analyses. In parallel, the culture medium was collected and the expression of IL-10 was determined via ELISA. All primers used for RT-qPCR were synthesized by life technologies (China), and their sequences are as follows (F: forward; R: reverse) (Table 3).

#### 3.3.3. Assessment of Anti-Inflammatory Effects of FE on Mice

This study was ethically approved by the Chinese Academy of Sciences, Guangzhou Biomedical and Health Research Institute, attributing the ethical approval code 2020070 on 10 April 2020. Male mice (C57BL/6) were obtained from the Animal Centre of Institute of Chinese Medical Sciences, University of Macau. All animals were raised in specific-pathogen-free animal rooms and treated according to the local policy for animal experiments. Male C57BL/6 mice (18–22 g) were randomly divided into three groups (*n* = 10 per group) and intraperitoneally injected with saline (Group I), LPS at 10 mg/Kg (Group II), and FE at 10 mg/Kg (30 min before LPS injection (Group III)). Eight hours after the injection, the mice were sacrificed, and the intraperitoneal macrophages were extracted by pump-backed cold PBS from the mouse abdomen, red blood cell lysis buffer was added and the cells were then washed twice with PBS. FITC anti-mouse F4/80 antibody and APC anti-mouse CD11C antibody were used for flow cytometric analysis; meanwhile; both the heart and lung organs were collected, embedded in paraffin, sectioned into 6 μm, and stained with hematoxylin and eosin (H&E).

### 3.4. Statistical Analysis

The result data were presented as the mean ± standard deviation (SD), from *n* ≥ 3. Statistical analyses were carried out using a one-way ANOVA (GraphPad Prism, San Diego, CA, USA), with *, ** and *** denoting *p* < 0.05, *p* < 0.01 and *p* < 0.001, respectively.

## 4. Conclusions

This study elucidated the anti-inflammatory behavior of a commercially available fucoidan extract (FE) at a pharmaceutical grade, obtained from *Fucus vesiculosus*. The results indicate that FE induced the expression of anti-inflammatory cytokines such as CD206 and IL-10 in BMDMs. The addition of FE reversed the increased expression of iNOs after LPS+IFN-γ stimulation, for values in the range of the control condition. The addition of FE increased CD206 and IL-10 expression in response to LPS+IFN-γ stimulation, proving the anti-inflammatory behavior. Regarding the in vivo studies, FE reversed inflammation after LPS stimulation, both the lung and cardiac tissues being normal when compared with the control group. In conclusion, the anti-inflammatory behavior of the commercial fucoidan extract evaluated in this study was confirmed, revealing that large-scale production could be further explored for application in clinical settings. Nevertheless, further studies are needed to better understand the structure–activity relationship, namely by investigating the chromatographic fractions of FE, with a narrower range of chemical features, and their anti-inflammatory activities.

## Figures and Tables

**Table 1:** Monosaccharide composition (% mol), total content of sugars, sulfation degree (% *w/w*), and molecular weight of fucoidan extract (FE).

	Monosaccharides (mol%)				Total Sugars (%, *w/w*)	Sulfate (%, *w/w*)	Mw (kDa)	Mw/Mn
	Fucose	Xylose	Galactose	Uronic Acids				
FE	90.4 ± 2.0	2.4 ± 0.7	3.3 ± 0.7	3.8 ± 0.7	66.1 ± 2.6	9.9 ± 2.9	70	1.5

**Table 2:** Methylation analyses before (native) and after the desulfation (desulfated) of FE.

Substitution	Native FE	Desulfated FE
t-Fuc	7.2 ± 0.3	10.8 ± 1.1
2-Fuc	6.7 ± 0.6	9.4 ± 0.3
3-Fuc	0.6 ± 0.0	3.8 ± 0.2
4-Fuc	3.4 ± 0.1	6.5 ± 1.0
2,3-Fuc	4.8 ± 0.4	5.6 ± 0.7
2,4-Fuc	9.9 ± 0.4	7.1 ± 0.5
3,4-Fuc	11.6 ± 0.7	16.8 ± 0.8
2,3,4-Fuc	49.9 ± 1.1	32.7 ± 2.6
Total Fuc	93.9 ± 1.2	92.7 ± 1.1
t-Xyl	4.0 ± 0.8	4.4 ± 0.5
2-Xyl	1.5 ± 0.1	2.0 ± 0.2
Total Xyl	5.5 ± 0.9	6.5 ± 0.7
t-Gal	0.6 ± 0.2	0.8 ± 0.4
Total Gal	0.6 ± 0.2	0.8 ± 0.4

**Table 3:** Primer sequences used for RT-qPCR procedures.

Gene	Forward (5′-3′)	Reverse (5′-3′)
TNF-α	ACGGCATGGATCTCAAAGAC	AGATAGCAAATCGGCTGACG
IL-12 p40	AGCAGTAGCAGTTCCCCTGA	AGTCCC TTTGGTCCAGTGTG
IL-10	GCTCTTACTGACTGGCATGAG	CGCAGCTCTAGGAGCATGTG
CD206	GCAGGTGGTTTATGGGATGT	GGGTTCAGGAGTGTTGTGG
iNOS	CCAAGCCCTCACCTACTTCC	CTCTGAGGGCTGACACAAGG
GADPH	AACGACCCCTTCATTGAC	TCCACGACATACTCAGCAC-3

## Data Availability

The data presented in this study are available in the main text.
